# BioEmu: AI‐Powered Revolution in Scalable Protein Dynamics Simulation

**DOI:** 10.1111/jcmm.70960

**Published:** 2025-11-24

**Authors:** Tianming Han, Min Wu, Qi Zhao

**Affiliations:** ^1^ School of Computer Science and Software Engineering University of Science and Technology Liaoning Anshan China; ^2^ EFREI Engineering School of Digital Technologies Paris‐Panthéon‐Assas University Villejuif France; ^3^ Wenzhou Institute University of Chinese Academy of Sciences Wenzhou China

In drug discovery and biotechnology, protein dynamics are essential for understanding functional mechanisms. However, traditional methods, such as molecular dynamics (MD) simulations, are time‐ and resource‐intensive, which often require supercomputers and months of computation. Recently, Lewis et al. [1] brought a giant advance in the journal of *Science*: BioEmu, a diffusion model‐based generative AI system. It simulates protein equilibrium ensembles with 1 kcal/mol accuracy using a single GPU, achieving a 4–5 orders of magnitude speedup for equilibrium distributions in folding and native‐state transitions. This approach reduces high computational costs and enables genome‐scale protein function prediction, akin to equipping biology with an accelerator.

Simulating protein dynamics is crucial for revealing functional mechanisms in drug development and biotechnology. Advances in deep learning tools, such as AlphaFold [2], enable genome‐scale prediction of protein sequences and static structures. However, quantitative analysis of dynamic equilibrium ensembles remains a bottleneck in this field [3]. Protein functions often emerge from transitions between conformational states and their probability distributions (Figure 1A) [4]. These transitions are regulated by factors like temperature, solvent, and concentration. Biophysical experiments, such as single‐molecule fluorescence or cryo‐EM, offer high precision but have low throughput and high costs [5]. MD simulations are versatile in principle, yet sampling challenges require massive computational resources. These often involve millisecond‐scale simulations, even with dedicated supercomputers or enhanced techniques. Prior to the Lewis et al. study, generative AI models could approximate protein shapes but failed to match experimental data precisely, especially for complex motions like domain rearrangements or cryptic pocket formation.

**FIGURE 1:**
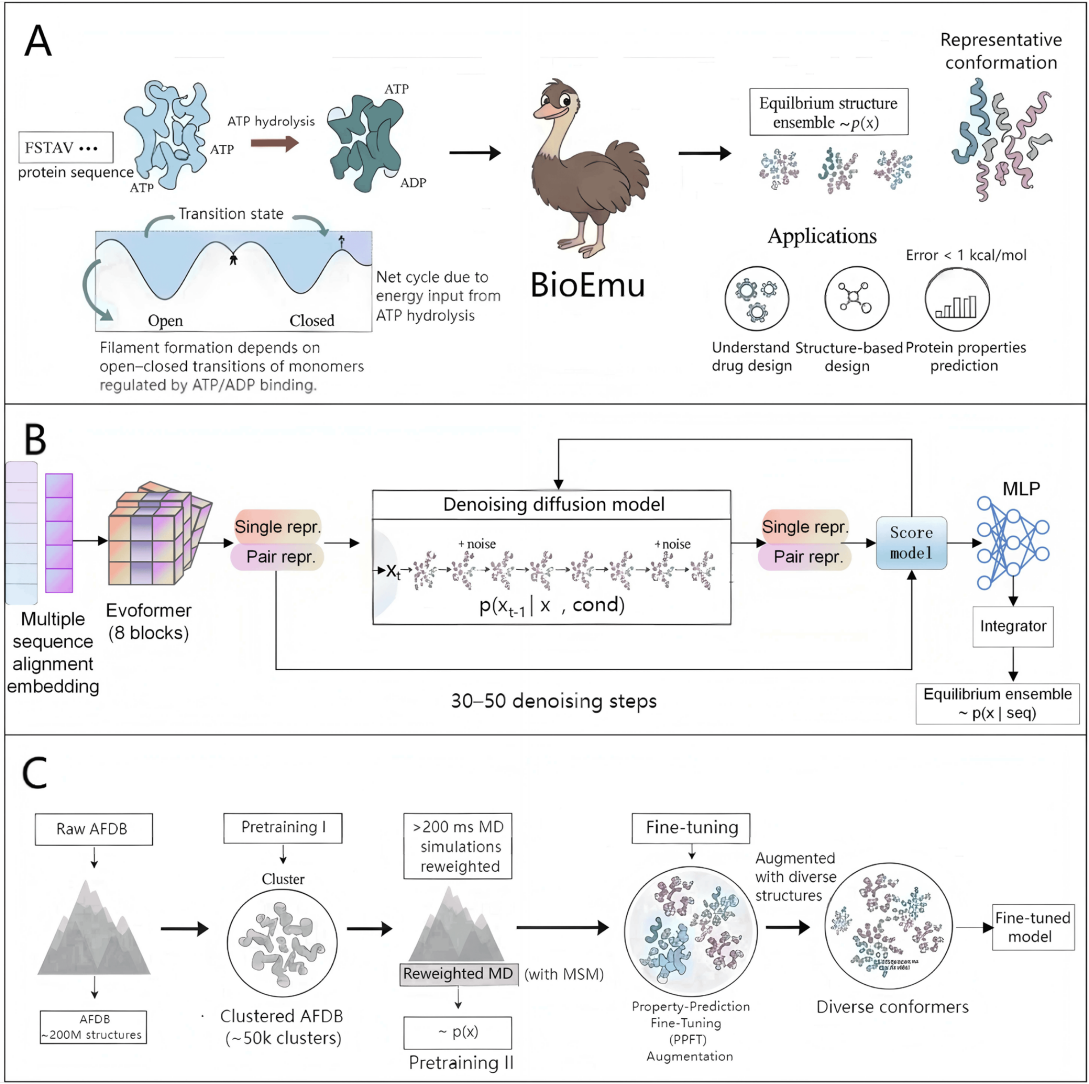
(A) Protein function via conformational changes (e.g., ATP hydrolysis driving filament formation) and high‐level BioEmu workflow, from sequence input to equilibrium ensembles and applications like structure‐based protein design and drug discovery. (B) Architecture, including sequence encoding, diffusion denoising (30–50 steps), and score models for ensemble generation. (C) BioEmu pretraining on clustered AFDB (~50 K clusters), continued training on > 200 ms MD simulations reweighted with MSM, and fine‐tuning with PPFT using > 500,000 MEGAscale stability measurements.

BioEmu's architecture combines protein sequence encoding with a generative diffusion model (Figure 1B). This system uses AlphaFold2's Evoformer module to convert the input sequence into single and pairwise representations. These representations capture deep associations between sequence and structure and are then fed into a diffusion‐based denoising model, which uses coarse‐grained backbone frames for protein structures to enhance computational efficiency. The diffusion process generates independent structural samples in 30–50 denoising steps on a single GPU. This design overcomes the sampling bottleneck of traditional MD simulations. As a result, BioEmu samples thousands of structures per hour on a single GPU, compared to months on supercomputing resources.

BioEmu's training comprises three stages (Figure 1C). First, it involves pretraining on a processed AlphaFold database (AFDB) with data augmentation to link sequences to diverse structures. This enhances the model's generalisation to conformational variations and prevents overfitting to static structures. Second, further training occurs on thousands of protein MD datasets totaling over 200 ms, reweighed using Markov state models (MSM) [6] for equilibrium distributions. Finally, property prediction fine‐tuning (PPFT) fine‐tunes the model on 500,000 experimental stability measurements from the MEGAscale dataset [7]. PPFT incorporates experimental observations (e.g., melting temperature) into diffusion training. It optimises the ensemble distribution by minimising discrepancies between predicted and experimental values. This ensures generated structures are diverse and thermodynamically constrained.

To evaluate the BioEmu's performance, Lewis et al. developed several benchmark datasets. These datasets emphasize out‐of‐distribution (OOD) generalization and distinct conformational states. Domain motions enable proteins to perform essential functions like enzyme catalysis, ligand binding, and signal transduction by allowing flexible rearrangements between conformational states, such as open‐closed transitions. In the domain motion benchmark, BioEmu effectively samples large‐scale open–closed transitions, covering reference experimental structures (RMSD ≤ 3 Å) with overall success rates of 55%–90% for known conformational changes. This performance surpasses baselines like AFCluster and DiG, which allow previously infeasible genome‐scale protein function predictions on a single GPU, revealing substrate‐induced free energy shifts and cryptic pockets for drug targeting. This platform is likely to accelerate drug discovery and biotechnology by reducing computation time from months to hours, though challenges remain with larger complexes.

The local unfolding benchmark assesses BioEmu's capacity to simulate flexible regions. The Switch II region of Ras p21 undergoes local unfolding upon GTP/GDP binding, whose change regulates cancer‐associated signalling pathways. BioEmu‐generated structures indicate the formation of a short α‐helix in the active state, but remain partially unfolded in the inactive state. These findings align with experimental data. BioEmu, by predicting the open states of cryptic pockets, also reveals drug‐binding sites that are hard to access in static structures, thereby accelerating drug design. In the sialic acid‐binding factor, this tool can uncover new sites for designing small‐molecule inhibitors to block sialic acid binding, weakening bacterial survival and particularly aiding the development of novel antibiotics against drug‐resistant strains. Meanwhile, in the Fascin protein, the open state exposes new binding sites, allowing the design of inhibitors to disrupt its bundling function and inhibit tumour cell migration and metastasis. Existing studies have developed anti‐metastatic inhibitors for Fascin, and BioEmu can quickly simulate dynamic pockets to optimise drugs (such as through virtual screening), which is especially valuable for hard‐to‐treat metastatic cancers, as traditional targets (like kinases) are already saturated. BioEmu may accurately predict pocket‐open states for the sialic acid‐binding factor and Fascin. Sampling success rates range from 55% to 80%. It outperforms models like AlphaFlow, particularly on OOD proteins, which appear more practical for unknown or complex proteins.

Thermodynamic accuracy in protein simulations refers to the ability to reliably predict free energy differences (ΔG) between conformational states, dictating the probabilities of those states at equilibrium. This is crucial because protein function often depends on rare transitions between states, influenced by temperature, solvation, and binding partners. High accuracy ensures that models are able to quantify stability and dynamics, bridging static structures from tools like AlphaFold to functional insights. Without this feature, predictions may miss low‐probability but biologically key states, leading to an incomplete understanding of mechanisms like enzyme catalysis or signalling. BioEmu shows exceptional thermodynamic accuracy in quantitative prediction tasks. PPFT fine‐tuning enables this by converting experimental stability data, such as melting temperature, into ensemble weights. This optimises sampling of low‐probability states. The authors examined how data scale affects accuracy. As total MD simulation time rises from 50 to 200 ms, prediction error drops linearly. This highlights the cost‐sharing strategy's effectiveness, where high initial data costs support numerous protein predictions.

The PPFT algorithm stands out as a key methodological innovation. It enables use of unstructured data via joint optimization of the property prediction head (MLP) and diffusion loss. Specifically, during fine‐tuning, it minimizes KL divergence between generated sample properties (e.g., ΔG) and experimental labels. This ensures thermodynamic consistency in the distribution. Unlike traditional supervised learning, this approach avoids overfitting and improves generalization to unseen sequences. In predicting protein stability, PPFT significantly enhances the model's ability to fit experimental data, highlighting its key role in optimizing ensemble distributions and thermodynamic accuracy.

The scarcity of thermodynamic constraints in previous protein generation models stems from several factors. First, data availability was limited: structural databases like the PDB typically provide single, static conformations without ensemble probabilities. Obtaining high‐throughput thermodynamic data (e.g., ΔG or ΔΔG) via experiments or long‐timescale MD simulations was computationally prohibitive. Second, methodological challenges arose in integrating physical scales, such as aligning model probabilities with Boltzmann distributions while maintaining equivariance. Third, evaluation norms favored geometric metrics (e.g., RMSD, TM‐score) over thermodynamic validation, which is more resource‐intensive. Recent advancements have enabled explicit thermodynamic calibration. These include large‐scale MD datasets, high‐throughput stability assays, mature equivariant diffusion/flow models, and growing demands in drug design and mutation prediction. BioEmu innovates by combining AlphaFold‐derived sequence representations with equivariant diffusion to generate sequence‐conditioned equilibrium ensembles. It calibrates these against extensive MD trajectories and experimental data, achieving less than 1 kcal/mol accuracy in relative free energy. This marks the first realization of thermodynamic scaling in full‐protein ensemble generation at high throughput on single GPUs. However, BioEmu primarily targets single‐chain proteins. Generalization to larger complexes (≥ 500 residues), multi‐chain systems, or long sequences may require further optimization. Integrating multimodal experimental data, such as cryo‐EM or single‐molecule fluorescence, poses additional challenges [8]. This highlights bottlenecks in sampling and generalization for larger systems [9]. The study indicates that diversifying training data through longer MD simulations and experimental observations can reduce prediction errors with increasing data scale, ultimately enhancing adaptability to complex systems. If validated, BioEmu could extend to multi‐chain macromolecules, enabling genome‐scale dynamics predictions and new drug target discovery.

In summary, Lewis et al. developed BioEmu as a scalable biomolecular simulator, which enables sampling of protein equilibrium ensembles. Hence, generative AI may accurately predict conformational changes, free energy distributions, and thermodynamic stability. This work establishes a framework for generating protein dynamics, which examines generative models from various perspectives and holds promise for advances in preclinical drug development and biomolecular function analysis.

## Author Contributions

**Tianming Han:** investigation, methodology, writing – original draft. **Min Wu:** conceptualization, methodology, writing – review and editing. **Qi Zhao:** conceptualization, funding acquisition, methodology, supervision, writing – original draft, writing – review and editing.

## Funding

This study is supported by the Science and Technology Plan Project of Liaoning Province (grant no. 2025‐MSLH‐351), and Fundamental Research Funds for the Liaoning Universities (grant no. LJ212410146026).

## Conflicts of Interest

The authors declare no conflicts of interest.

## Data Availability

Data sharing not applicable to this article as no datasets were generated or analysed during the current study.
